# Diurnal Variation and Twenty-Four Hour Sleep Deprivation Do Not Alter Supine Heart Rate Variability in Healthy Male Young Adults

**DOI:** 10.1371/journal.pone.0170921

**Published:** 2017-02-02

**Authors:** Daniel S. Quintana, Torbjørn Elvsåshagen, Nathalia Zak, Linn B. Norbom, Per Ø. Pedersen, Sophia H. Quraishi, Atle Bjørnerud, Ulrik F. Malt, Inge R. Groote, Tobias Kaufmann, Ole A. Andreassen, Lars T. Westlye

**Affiliations:** 1 NORMENT, KG Jebsen Centre for Psychosis Research, Division of Mental Health and Addiction, University of Oslo, and Oslo University Hospital, Oslo, Norway; 2 Department of Neurology, Oslo University Hospital, Oslo, Norway; 3 Institute of Clinical Medicine, University of Oslo, Oslo, Norway; 4 Department of Psychology, University of Oslo, Oslo, Norway; 5 Barnard College, Columbia University, New York, New York, United States of America; 6 The Intervention Centre, Oslo University Hospital, Oslo, Norway; 7 Department of Physics, University of Oslo, Oslo, Norway; 8 Department of Research and Education, Division of Clinical Neuroscience, Oslo University Hospital, Oslo, Norway; University of Lübeck, GERMANY

## Abstract

Heart rate variability (HRV) has become an increasingly popular index of cardiac autonomic control in the biobehavioral sciences due to its relationship with mental illness and cognitive traits. However, the intraindividual stability of HRV in response to sleep and diurnal disturbances, which are commonly reported in mental illness, and its relationship with executive function are not well understood. Here, in 40 healthy adult males we calculated high frequency HRV—an index of parasympathetic nervous system (PNS) activity—using pulse oximetry during brain imaging, and assessed attentional and executive function performance in a subsequent behavioral test session at three time points: morning, evening, and the following morning. Twenty participants were randomly selected for total sleep deprivation whereas the other 20 participants slept as normal. Sleep deprivation and morning-to-night variation did not influence high frequency HRV at either a group or individual level; however, sleep deprivation abolished the relationship between orienting attention performance and HRV. We conclude that a day of wake and a night of laboratory-induced sleep deprivation do not alter supine high frequency HRV in young healthy male adults.

## Introduction

Heart rate variability (HRV) provides a non-invasive measure of cardiac parasympathetic nervous system (PNS) control [1, 2]. Meta-analyses have established a relationship between poor cardiac autonomic regulation and a range of psychiatric disorders [3–6]. HRV has also been associated to specific psychiatric illness symptoms, such as social cognition [7, 8], executive function [9], and self-regulation [10]. Given the association with psychiatric symptoms across diagnoses, researchers have proposed that HRV may be a transdiagnostic symptom biomarker [11]. HRV is also fundamental to leading biobehavioral frameworks used to understand social behavior and psychiatric illness, the polyvagal theory [12] and the neurovisceral integration model [13]. However, to become a viable biomarker in studies of psychopathology, its intraindividual stability and variables that can modulate HRV need to be established.

HRV has demonstrated good-to-excellent reliability from day-to-day [14], week-to-week [15, 16], and month-to-month [17, 18]. Nevertheless, the impact of diurnal and sleep quality variability is not well understood as their reported effects on HRV are mixed. For instance, after 24 to 26 hours of sleep deprivation, some researchers have reported no significant group changes in HRV [19, 20], whereas greater periods of deprivation (i.e., 36–60 hours) have been associated with decreases in HRV [21–25], suggesting that longer periods are more likely to elicit effects on HRV. Relatedly, reports on the effects of sleep deprivation on heart rate (HR), which represents a mixture of parasympathetic and sympathetic outflow, have also been variable between studies [20, 26, 27]. Notwithstanding these mixed results, it is also not known whether sleep deprivation and diurnal changes influence HRV on an individual level, particularly in populations more susceptible to sleep dysfunction, such as those with psychiatric illness [28, 29] and cardiovascular diseases [30, 31]. These populations are also more prone to systemic inflammation and insulin resistance [32–35], which are also related to both vagal function [36, 37] and sleep deprivation [38, 39]. Relatedly, sleep deprivation also impairs performance on cognitive tasks, such as decision-making, impulsivity, and attention [40–42]. The neurovisceral model proposes that HRV is related to the regulation of goal-directed behaviors [13], which is typically impaired in psychiatric illness [43, 44]. While HRV has been reported to be related to executive function performance [7, 45], research has yet to determine the relationship between ANS regulation and attentional executive function performance, and how this relationship is affected by sleep deprivation and diurnal variation. Therefore, the aim of this study was to examine the impact of diurnal variation and 24-hour sleep deprivation on HRV and attentional executive function in a homogenous sample of healthy male young adults.

## Materials and Methods

### Participants

Forty adult males (mean age = 22.13, SD = 2.44, range = 18–26) were recruited to participate in the study and were randomly assigned either to a sleep deprivation group (*n* = 20) or a sleep group (*n* = 20). Magnetic resonance imaging (MRI) data from this study has been previously described [46, 47]. Exclusion criteria assessed by clinician interview included history of sleep disorder, neurological or other chronic somatic disorder (e.g., cardiovascular and metabolic diseases), current acute somatic illness, psychiatric illness, use of psychotropic drugs, alcohol or drug use disorder, head injury with loss of consciousness for more than one minute, and metallic implants. Participants self-reported regular sleep-wake cycles comparable to a large Norwegian dataset of 23 year olds (n = 560) [48]. The present study received approval from the Regional Committee for Medical Research Ethics. After receiving information regarding the study, participants provided written informed consent.

### Physiology data collection and analysis

The current study was reported in accordance with the Guidelines for Reporting on Articles on Psychiatry and Heart rate variability (GRAPH) [49], which provides a standardized set of criteria for reporting HRV studies in the biobehavioral sciences (S1 Appendix). HRV via pulse oximetry data from the participants was collected during MRI sessions in the morning after a self-reported night of regular sleep in their homes, the same evening, and then the next morning. The average times and time ranges of data collection are presented in Table 1. A self-reported average of hours of sleep over the previous month was also collected. The time of the first data collection was adjusted to the participants' usual sleep–wake cycles, which were determined by self-report. The average time between data collection during first morning and the night was approximately 14 hours and between each morning was approximately 23 hours. No intake of caffeine, nicotine, or alcohol was allowed from the night before the study day until study completion and no intake of food or energy-containing fluids was allowed the 3 hours before each MRI session. Otherwise, no restrictions were placed on fluid or food intake before or during study participation. After the first visit, participants were instructed not to sleep and to refrain from physical activity, otherwise perform their regular daily activities, and returned at 9PM the same evening for the second data collection. After the evening examination, the sleep deprivation group stayed overnight at the hospital and was continuously monitored by a research assistant to ensure that none fell asleep. Participants played video games, read books, or watched movies during this overnight period. The sleep group left the hospital after the second MRI session, had a night of sleep in their homes and returned to the hospital the next morning for the final data collection.

**Table 1:** Data collection times.

Data collection period	Sleep (n = 20)	Sleep-deprived (n = 20)
Morning 1	08:07 (6:40–9:25)	08:22 (7:30–9:45)
Night 1^a^	21:28 (20:30–22:15)	22:31 (21:10–23:50)
Morning 2^b^	07:54 (6:45–9:45)	06:30 (05:25–07:20)

Note. 24-hour time is presented. Range of time is presented in parenthesis.

^a^ Sleep group = 19, sleep-deprived group = 19

^b^ Sleep group = 18, sleep-deprived group = 18.

To help control for light exposure during sleep deprivation, the sleep-deprived individuals remained in the same room with constant, normal light intensity between the evening and the second morning examinations. The 5 x 4-meter windowless room was furnished with chairs, a table, and a TV. During the night, the participants were reading books, talking with the research assistant, playing video games, and watching movies. The total amount of light exposure from the TV of each participant was not assessed.

Pulse-to-pulse intervals–an approximation of beat-to-beat intervals–were measured from 7 minutes of pulse oximetry data collected during both resting functional MRI and arterial spin labelling from a photoplethysmograph placed on the right index finger (50Hz). A photoplethysmograph offers an accessible alternative to ECG for collecting HRV data in the MRI environment, as ECG is susceptible to significant interference from MRI sequences. Participants were instructed to lie still with their eyes open during the scan. The first two minutes of IBI data were discarded for all participants to account for any differences in habituation to the imaging procedure, thus a 5-minute sample was used for analysis. While pulse interval is not a direct interbeat interval measure as it is derived from both the time between pulse wave initiation (i.e., SA node firings) and the changes in pulse transit time, this data offers a very accurate approximation of interbeat intervals during immobility [50, 51], but poorer performance during exercise [52]. As per recommendations [2], raw data were upsampled using spline interpolation to 1000Hz to refine the R-wave fiducial point for HRV calculation in ARTiiFACT [53]. Artifacts were detected using an algorithm by Berntson and colleagues [54]. Any detected artifacts were manually checked, with the rater blind to participant group. Estimated intervals using cubic spline interpolation replaced detected artifacts. Absolute high frequency (HF; 0.15–0.4 Hz) power, which indexes cardiovagal activity [2], was calculated to assess HRV using the Fast Fourier Transformation (FFT). The FFT applied a Hanning window of 256-s width with an interpolation rate of 4Hz (spline interpolation) and an overlap of 50% to the resampled and detrended data (method of least squares). Finally, absolute HF values were log transformed to better approximate a normal distribution. HRV data from both resting functional MRI and arterial spin labelling recordings were averaged to increase reliability of the HRV calculation. Thirteen data points (out of 120) were not available for analysis due to equipment malfunction (morning 1: sleep group n = 1, sleep-deprived group n = 3; night 1: sleep-deprived group n = 3; morning 2: sleep-deprived group n = 3).

### Attention network task

Participants completed the attention network task (ANT; [55]) to assess vigilance and attentional brain networks approximately five minutes after HRV data collection while seated at all three time points, which provided enough time for stabilisation of the hemodynamic response to posture change [56]. We did not measure HRV during the task. Regardless, our primary interest was the relationship between task performance and HRV during specific states (i.e., sleep-deprived vs. rested). By comparing response times (RTs) between different conditions, ANT is often used to compute behavioral indices of attentional alerting, orienting, and conflict inhibition (i.e., executive control). Alerting refers to the achievement and maintenance of vigilance to stimuli, orienting to the selection and orienting to stimuli, and conflict inhibition to the ability to resolve incongruent stimuli [57]. RT was recorded in three different conditions by flankering target stimuli with congruent, incongruent or neutral stimuli, which were preceded by no cue, double-cue, or a single valid cue. The alerting, orienting, and conflict inhibition components of the ANT were defined by comparing the RT distribution in specific task conditions, as previously described [58], yielding t-values for each behavioral component. The average RT for correct trials across all conditions was also calculated.

### Statistical analysis

All statistical tests were conducted using the R statistical software package [59] and the JASP statistical package [60]. Continuous demographic variables were compared via Welch’s *t*-test. Repeated measures ANOVA using both frequentist and Bayesian statistics (Jeffreys-Zellner-Siow Bayes factor with default prior scales) were used to assess the main effects of experimental group, time, and their interaction on HRV, HR, and ANT measures [61]. Bayes factors (BF) provide evidence for a specified null hypothesis against a specified alternative hypothesis. Importantly, BFs can also quantify evidence for the null hypothesis against the alternative hypothesis, which is not possible using a frequentist approach. A BF value less than 0.33 provides substantial evidence for the null hypothesis, over 3 provides substantial evidence for the alternative hypothesis and between 0.33 and 3 provides no strong support either way, suggesting an underpowered study [62]. The concordance correlation coefficient (CCC) was also calculated for comparisons of HRV [63] using the “agRee” R package (http://CRAN.R-project.org/package=agRee). A CCC of 0 represents perfect disagreement whereas 1 represents perfect agreement. ANT measures (i.e., alerting, orienting, and conflict inhibition) were correlated with HRV at baseline for all participants. Pearson correlation tests were performed to assess the relationship between ANT measures and HRV in the first and the second morning in the sleep and sleep deprivation groups. The probability that these correlations are different was computed by examining the posterior difference of the Bayes correlation tests. Finally, frequentist and Bayesian hierarchical regression was used to assess the impact of subjective sleepiness, as measured using the Karolinska Sleepiness scale [64], on the relationship between HRV and ANT responses.

## Results

**Fig 1:**
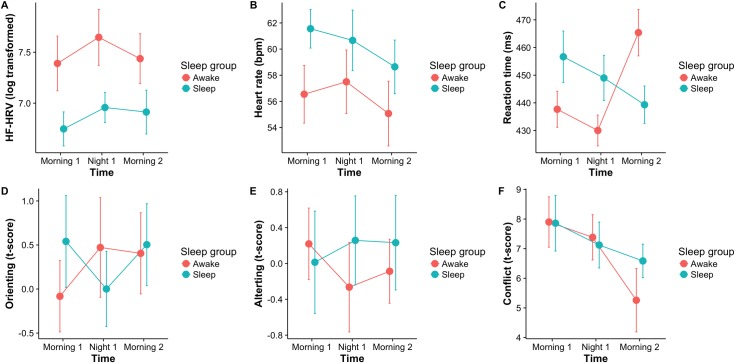
Main effects and interactions of group and measurement period on heart rate variability, heart rate, and ANT measures. Line graphs illustrate means and 95% confidence intervals for log-transformed HF-HRV (1A), HR (1B), mean reaction time (1C), Orienting t-values (1D), Alerting t-values (1E), and Conflict t-values (1F). Error bars represent 95% confidence intervals, which are corrected to remove between-subject variability. M1 = Morning 1; N1 = Night 1, M2 = Morning 2.

Demographics, sleep, and heart rate variables are summarized in Table 2. There were no significant differences in age, sleep habits, or mood variables between the sleep and sleep deprivation groups (Table 2). While participants had less hours of sleep the night previous to testing (mean = 6.75, SD = 1.2) compared to the average hours of sleep over the previous month (mean = 7.61, SD = 0.8; *t*(39) = -4.37, *p* < 0.001), there was no difference in hours of sleep the previous night between groups (Table 2). Between-subjects ANOVA revealed a main effect for group on HRV, with the data providing substantial support that the sleep-deprived group had higher HRV compared to the sleep group (Table 3, Fig 1A). There was no main effect of time or time by group interaction, with the BFs providing substantial evidence for the null hypotheses that HRV was not different across time and that there were no group by time interaction effects on HRV. As the BF for the time by group interaction was 0.19, this indicates that the data were appropriately powered and the null hypothesis was 5.3 more likely than the alternative hypothesis. Between-subjects ANOVA revealed no significant main effect for group on HR–with data providing anecdotal support that the sleep-deprived group had a decreased HR overall compared to the sleep group–and no main effect of time (Table 3, Fig 1B). Finally, there was no significant time by group interaction, with data providing substantial evidence for the null hypothesis that there was no group by time interaction effects on HR (Table 3, Fig 1B).

**Table 2:** Demographic, sleep, and heart rate variability at baseline.

Group	Total (n = 40)	Sleep (n = 20)	Sleep-deprived (n = 20)	*t* (df)	*p*
Age	22.1 (2.4)	22.7 (2.1)	21.6 (2.7)	-1.5 (36.3)	0.14
HRV ^a^	7.1 (1)	6.8 (1)	7.4 (0.8)	2.1 (33.8)	0.04
Heart rate (bpm) ^a^	59.2 (8.8)	61.6 (10.3)	56.6 (6)	-1.8 (29.3)	0.08
Karolinska sleepiness scale	3.8 (1.7)	3.8 (1.7)	3.9 (1.7)	0.3 (38)	0.78
Hours of sleep previous night	6.8 (1.2)	6.6 (1)	6.9 (1.4)	0.6 (34.6)	0.53
Sleep quality previous night VAS	49.8 (24.1)	45 (27.6)	54.6 (19.5)	1.3 (34.2)	0.21
PSQI score ^b^	4.3 (2.1)	4.6 (2.5)	4 (1.7)	-0.9 (31.3)	0.36
Stress VAS	23.5 (17.8)	26.6 (20)	20.5 (15.3)	-1.1 (35.5)	0.28
Depression previous month VAS	12 (15.6)	10.4 (15.5)	13.6 (15.9)	0.6 (38)	0.53
Anxiety previous month VAS	11 (14.2)	6.9 (12.9)	15.1 (14.5)	1.9 (37.5)	0.07
Alcohol units consumed previous month	13 (15.9)	14.4 (18.9)	11.7 (12.6)	-0.5 (33.1)	0.59
Hours of physical activity previous week	4.4 (4)	4.8 (3)	3.9 (4.8)	-0.7 (31.7)	0.47

Note. Group means compared using Welch's t-test, values are means with standard deviations in parenthesis. bpm = beats per minute; HRV = Heart rate variability, log transformed absolute high frequency power; PSQI score = Pittsburgh sleep quality index total score; VAS = visual analogue scale.

^a^ sleep group n = 17, sleep-deprived group n = 19.

^b^ sleep group n = 19.

**Table 3:** The impact of sleep deprivation on HRV and HR.

	Between-subjects ANOVA			
	*df*	*F*	*P*	BF
HRV				
Time	2, 68	1.7	0.19	0.32
Group	1, 34	6.75	0.01	3.8
Time and Group interaction	2, 68	0.29	0.75	0.19
HR				
Time	2, 68	2.31	0.11	0.56
Group	1, 34	3.67	0.06	1.51
Time and Group interaction	2, 68	0.06	0.94	0.16

Considering the observed differences at baseline, independent samples t-tests were also performed comparing the percentage change in HRV and HR from morning-to-morning between groups. These tests revealed similar results, with no significant percentage change difference between groups in HRV (*t*(23.55) = 0.71, *p* = 0.49, Cohen’s *d* = 0.24, BF = 0.4) or HR (*t*(27.44) = -0.03, *p* = 0.97, Cohen’s *d* = 0.01, BF = 0.32). Considering the recognized effects of respiration on HRV [65], the effect of time and group on peak HF frequency was calculated to approximate respiratory frequency. Analysis revealed no statistically significant main effect of time (*p* = 0.25, BF = 0.23), group (*p* = 0.34, BF = 0.46), or time by group interaction (*p* = 0.12, BF = 0.72). There also was no association of the time between morning sessions and HF-HRV change in the sleep [*r* = 0.01, 95% CI (-.46, .48), *n* = 19, *p* = .95], or sleep-deprived [*r* = 0.26, 95% CI (-.29, .68), *n* = 17, *p* = .35] groups, which suggests that any differences in time between morning measurements were unlikely to influence changes in HRV.

**Fig 2:**
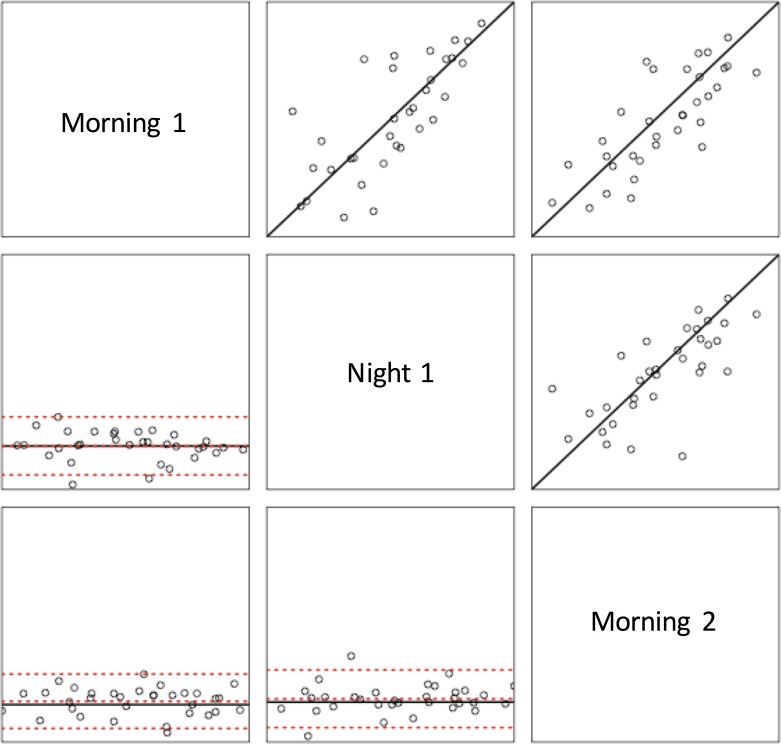
A matrix of plots illustrating the agreement of HRV between three time points. The upper right panels consist of scatterplots with identity line (45° line though the origin). The lower left panels consist of Bland-Altman plots with confidence bounds and bias (dotted red line) and the horizontal black line passing through the origin. The confidence bounds show the mean of the difference between time points plus or minus twice of the standard deviation of the difference.

The CCC point estimate comparing HRV at morning and night was 0.76 [95% CI (.55, .88)], which suggests good reproducibility between sampling points. Relatedly, the Pearson correlation coefficient was 0.77 [95% CI (.59, .88), *n* = 36, *p* < .001], indicative of a strong association between HRV collected in the morning and night. The CCC point estimate comparing the reproducibility of HRV for all three time points was 0.77 [95% CI (.62, .86)], which suggests good reproducibility between all three sampling points (Fig 2). There was also a significant relationship between the two morning visits [*r* = 0.8, 95% CI (.64, .89), *n* = 36, *p* < .001; Fig 2] and the night and second morning visits [*r* = 0.77, 95% CI (.59, .87), *n* = 37, *p* < .01; Fig 2] across all participants.

There was no main effect of group on ANT reaction time (Fig 1C), but a significant main effect for time for ANT reaction time and a strong interaction of group and time (Table 4), indicating slower responses during the second morning in the sleep deprivation group. There were no interactions or main effects for orienting (Fig 1D) or alerting (Fig 1E). There was a main effect for time for conflict inhibition (Fig 1F), suggestive of a learning effect for conflict inhibition effect, regardless of group, but no main effect for group, or a time by group interaction (Table 4). Analysis revealed a significant correlation between HF-HRV and orienting at baseline across participants (Table 5). Examining each group separately at baseline revealed similar coefficients.

**Table 4:** Attention network task performance.

	Repeated-measures ANOVA			
	*df*	*F*	*P*	BF
Reaction time				
Time	2, 76	6.39	0.003	1.8
Group	1, 38	0.13	0.72	0.45
Time and Group interaction	2, 76	25.83	< 0.001	> 10000
Orienting				
Time	2, 76	1.94	0.17	0.18
Group	1, 38	1.08	0.39	0.27
Time and Group interaction	2, 76	1.85	0.18	1.64
Alterting				
Time	2, 76	0.14	0.87	0.1
Group	1, 38	0.75	0.39	0.26
Time and Group interaction	2, 76	1.34	0.27	0.38
Conflict inhibition				
Time	2,76	12.34	< 0.001	26.31
Group	1,38	2.29	0.11	0.51
Time and Group interaction	2,76	0.28	0.6	0.74

**Table 5:** The relationship between ANT scores and HRV.

		Baseline (n = 36)	Sleep (n = 20)	Sleep deprivation (n = 20)
Orienting	Pearson's r	-0.39	-0.55	-0.13
	p-value	0.02	0.01	0.62
	Upper 95% CI	-0.07	-0.16	0.37
	Lower 95% CI	-0.64	-0.8	-0.57
Alerting	Pearson's r	0.05	-0.01	-0.07
	p-value	0.79	0.98	0.79
	Upper 95% CI	0.37	0.44	0.42
	Lower 95% CI	-0.29	-0.45	-0.53
Conflict inhibition	Pearson's r	0.08	0.27	-0.07
	p-value	0.62	0.24	0.8
	Upper 95% CI	0.4	0.63	0.43
	Lower 95% CI	-0.25	-0.19	-0.53

**Fig 3:**
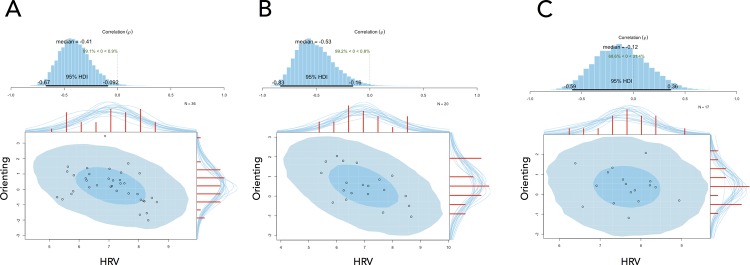
The relationship between orienting and HRV. Plots demonstrate the relationship between orienting and HRV at baseline (3A) after sleep (3B) and after sleep deprivation (3C). The blue histogram shows the posterior distribution for the correlation *p* with a 95% highest density interval (HDI). The scatterplots illustrate the relationships between these two variables, with superimposed posterior predictive distributions. The larger light blue ellipse shows the 95% highest density region while with smaller dark blue ellipse shows the 50% highest density region. The histograms on the top x-axes and right y-axes show the marginal distributions of the data. HDI = Highest density interval.

A significant relationship between HF-HRV and orienting was also observed the next morning in the sleep group, however, there was no significant relationship in the sleep-deprived group (Table 5). A Bayesian Pearson correlation test revealed an estimated correlation (*p*) of -0.41 between HF-HRV and orienting at baseline for all participants [95% CI (-.67, -.09), n = 36; Fig 3A], suggesting that the correlation coefficient is less than 0 by a probability of 99.1%. The corresponding correlation (*p*) for the sleep group during the second morning was -0.53 [95% CI (-.83, -.16), n = 20; Fig 3B], suggesting that the correlation coefficient is less than 0 by a probability of 99.2%. The corresponding correlation (*p*) for the sleep-deprived group during the second morning was -0.12 [95% CI (-.59, .36), n = 17; Fig 3C], indicating that the correlation coefficient is less than 0 by a probability of 68.6%. Computing the posterior difference of *p* between the sleep and sleep-deprived group Bayesian correlation tests revealed a 90.1% probability that *p* was more negative in the sleep group compared to the control group. There were no significant relationships observed between HRV and alerting or conflict (Table 5). Finally, frequentist and Bayesian hierarchical regression revealed that sleepiness did not contribute to the relationship between ANT responses and HRV at baseline (S1 Table) or second morning recordings (S2 and S3 Tables).

## Discussion

Utilizing a repeated-measures design, we have demonstrated that supine HRV is robust against 24-hour sleep deprivation and diurnal variation in healthy male young adults. As well as providing substantial evidence for no significant group by time interaction on HRV, the data also indicate strong reproducibility of HRV measures on an individual level across both sleep deprived and non-sleep-deprived participants. Consistent with the neurovisceral integration model [13], the data suggest that HRV is related to orienting attention at baseline; however, this relationship was not evident after sleep deprivation. The present research is also congruous with evidence suggesting that PNS, which is closely approximated by HRV, is not influenced by diurnal factors [66] or sleep disturbances [20], at least for a 24-hour period. It has been previously suggested that the effect of sleep deprivation on the ANS may only manifest in those who are more susceptible to the psychological stressors associated with sleep deprivation [19]. Thus, the low likelihood of either trait or state anxiety in the study participants may have contributed to the non-significant effect of sleep deprivation on HRV. Reductions in HRV observed in longer periods of sleep deprivation [21–25] indicate that the PNS may only be robust against shorter periods of deprivation. Moreover, circadian influences on HRV have been reported [20, 67–69], however, in these instances HRV was recorded more regularly throughout the day. Regular assessment provides a much more nuanced view of circadian rhythms than the comparison of morning-to-night diurnal differences, thus the divergence of present result with other research may be due to having only one time point comparison. Moreover, studies investigating circadian changes tend to report increased HRV during the day [67], whereas the current study only recorded data at morning and night. Thus, researchers should still be particularly vigilant when comparing HRV collected during the afternoon to morning and night HRV data.

Interest in biological rhythms within biobehavioral neuropsychiatric research has accelerated since the introduction of the Research Domain Criteria [70]. Typically, this approach targets potential dimensions of biological markers of psychiatric symptoms, which cut across diagnostic categories. Although there is growing evidence for biological rhythms impairments in psychiatric illness [71], the present data indicates that shorter periods of sleep deprivation and dysfunction do not influence HRV in healthy participants. Polyvagal theory suggests that behaviour can be facilitated by outflow of the vagus nerve, with a reduction of vagal outflow associated with threat-related behaviours [12]. Given that participants were kept as comfortable as possible, the experimental design was an approximate surrogate of chronic sleep deprivation, which tends to be chronic and associated with mood disorders and psychological distress [72]. Consequently, although participants had the physiological exposure of sleep-deprivation, they probably did not experience the adverse psychological aspects often associated with sleep deprivation in a natural setting, which may explain why reductions in HRV were not observed in the sleep-deprivation group. Further studies on the long-term effects of sleep deprivation on indices of cardiovascular functioning are needed, preferably combining the rigorous control afforded by experimental studies and the ecological validity provided by naturalistic studies.

Respiration is deeply integrated with ANS physiology and has a direct influence on HRV [73, 74], with increased frequency associated with reduced HRV due to lengthening of the heart period [75]. Although a study limitation was that a direct measure of respiration was not collected during PRV recordings, it is generally accepted that control for respiration is not required during non-task recordings in repeated-measures designs, at least in healthy participants, as respiratory frequency is not related to HRV [76, 77]. Research suggests that 24-hour sleep deprivation does not change resting ventilation patterns [78, 79] and we did not find a statistically significant time by group interaction for the HF peak frequency (a proxy of respiratory frequency), however, we cannot rule out the possibility that sleep deprivation influenced respiratory depth or other respiratory patterns not captured by HF peak frequencies. Relatedly, we did not measure HRV during the ANT task. Although this may have introduced task-related respiratory confounds [73], vigilance and attention levels are likely to be associated with respiration rate. Sleep-deprived individuals are likely to have less capacity for vigilance and attention, which could be exacerbated by increased respiratory rates. Indeed, slowing the respiratory rate decreases parasympathetic withdrawal in response to threat [80] and expiration tends to be shorter under the experimental induction of fear [81]. Thus, with the present design we cannot rule out the influence of respiratory patterns on vigilance and attention and how this is related to HRV.

Additional study limitations include the generalizability of the findings. Firstly, while participants were supine and immobile, the present data cannot be strictly considered “resting-state”, as the MRI environment is confined and exceptionally loud. Second, the present study used pulse oximetry data to calculate HRV, rather than the more common approach of using ECG. This limits direct comparison with other sleep deprivation HRV studies that use ECG. Moreover, while pulse-to-pulse HRV methods are comparable to gold standard approaches, using ECG and a higher raw sampling rate could have improved data accuracy [2]. Third, the study was limited to healthy young male adults. Fourth, as the ANS functions differently when the body is supine and upright [56], we can only extrapolate from the data how supine HRV is related to cognitive performance while seated. However, postural differences for HF-HRV may be less pronounced for a shift to a seated position compared to a shift to a standing position [82]. Thus, to improve generalizability, future research would benefit from recruiting a more representative population to collect ECG data at rest using the same posture when collecting cognitive data.

Since information about the participants’ sleep-wake cycles was determined by self-report and not obtained objectively (e.g., using actigraphy or polysomnography), we cannot rule out that participants were examined at different times of their circadian phases and that this might have affected the HRV data. Moreover, to control for light exposure during sleep deprivation, all sleep-deprived individuals stayed in a room with constant illumination between the evening and the next morning examinations. However, we did not control for the amount of light exposure from screens (e.g., television) that the participants used during sleep deprivation. Although more research on the effects of light exposure on HRV during sleep deprivation is needed, several studies compared the effects of bright and dim light exposure on indices of parasympathetic activity at rest and found no differences [83, 84]. Thus, it is possible but unlikely that the potentially varying light exposure from the TV among our participants substantially affected their HF-HRV data. Additionally, while both groups were exposed to the same MRI environment, it is possible that this could have reduced any differences in HRV between the experimental groups. Nonetheless, by the third scan (in which the greatest differences in HRV were expected) the participants would have habituated to the MRI environment given the past two scans within the same 24-hour period. Finally, although there was no significant time x group interaction, these unexpected baseline group differences may have contributed to the results, making data interpretation more complex.

In summary, this study revealed that sleep deprivation does not modulate the difference in HRV between three time points over a 24-hour period. This suggests that HRV is a robust measure that does not appear to be influenced by 24-hour sleep deprivation in an experimental environment, at least in healthy male young adults. While biological systems such as the brain functional connectome [46] and the metabolome [85] are modulated by sleep deprivation, the PNS appears to avoid these effects (at least with 24-hour sleep deprivation). If HRV is robust against sleep and diurnal disturbances, as the data suggest, this would reduce the burden on the researcher and participant as fewer resources are required to control for time of day effects (for morning vs. night recordings) and sleep habits. Future research is required to investigate if this is also observed in populations with reduced PNS activity, such as individuals with psychiatric illnesses, in females, and in older individuals.

## Supporting Information

S1 Appendix Guidelines for reporting articles on psychiatry and heart rate variability (GRAPH) checklist items. (PDF)

S1 Dataset Study data. (XLS)

S1 Table Hierarchical regression model comparisons for morning 1. (XLSX)

S2 Table Hierarchical regression model comparisons for morning 2 (sleep deprived group). (XLSX)

S3 Table Hierarchical regression model comparisons for morning 2 (sleep group). (XLSX)
